# Evaluation of two methods for computational HLA haplotypes inference using a real dataset

**DOI:** 10.1186/1471-2105-9-68

**Published:** 2008-01-29

**Authors:** Bruno F Bettencourt, Margarida R Santos, Raquel N Fialho, Ana R Couto, Maria J Peixoto, João P Pinheiro, Hélder Spínola, Marian G Mora, Cristina Santos, António Brehm, Jácome Bruges-Armas

**Affiliations:** 1 Hospital Santo Espírito de Angra do Heroísmo, SEEBMO, Angra do Heroísmo, Azores, Portugal; 2 Genetics & Arthritis Research Group (GARG), Institute for Molecular and Cell Biology (IBMC), University of Porto, Porto, Portugal; 3 University of Madeira, Campus of Penteada, Funchal, Madeira, Portugal; 4 Hospital Universitário de Canarias, Tenerife, Canarias, Spain; 5 Center of Research in Natural Resources (CIRN) and Department of Biology, University of The Azores, Campus of Ponta Delgada, Ponta Delgada, Azores, Portugal

## Abstract

**Background:**

HLA haplotype analysis has been used in population genetics and in the investigation of disease-susceptibility locus, due to its high polymorphism. Several methods for inferring haplotype genotypic data have been proposed, but it is unclear how accurate each of the methods is or which method is superior. The accuracy of two of the leading methods of computational haplotype inference – Expectation-Maximization algorithm based (implemented in Arlequin V3.0) and Bayesian algorithm based (implemented in PHASE V2.1.1) – was compared using a set of 122 HLA haplotypes (A-B-Cw-DQB1-DRB1) determined through direct counting. The accuracy was measured with the Mean Squared Error (*MSE*), Similarity Index (*I*_*F*_) and Haplotype Identification Index (*I*_*H*_).

**Results:**

None of the methods inferred all of the known haplotypes and some differences were observed in the accuracy of the two methods in terms of both haplotype determination and haplotype frequencies estimation. Working with haplotypes composed by low polymorphic sites, present in more than one individual, increased the confidence in the assignment of haplotypes and in the estimation of the haplotype frequencies generated by both programs.

**Conclusion:**

The PHASE v2.1.1 implemented method had the best overall performance both in haplotype construction and frequency calculation, although the differences between the two methods were insubstantial. To our knowledge this was the first work aiming to test statistical methods using real haplotypic data from the HLA region.

## Background

The Human Leukocyte Antigen (HLA) is a highly polymorphic gene cluster located on the Major Histocompatibility Complex (MHC) on the chromosome 6 (6p21) [1]. The MHC has an extension of 3.5 megabases and contains over 200 genes divided into three sub-regions: Class I (1800 kb), Class II (800 kb) and Class III (1100 kb) [1]. The HLA genes are the most polymorphic group known in the human genome. Their high rate heterozygosity is the result of the presence of three genes encoding classical MHC Class I and three or four gene sets for the classical MHC Class II molecules, on each chromosome. The cells express at least three different MHC proteins, decreasing the chance of each individual being homozygous at all these three loci [2]. Another important feature of HLA genes is the presence of Linkage Disequilibrium (LD) between the alleles of the different loci. Despite the large number of alleles at each expressed loci, the number of haplotypes observed in populations is smaller than the expected. This fact indicates that certain HLA alleles tend to occur together in the same haplotype rather than randomly segregating together [3,4].

HLA haplotype analysis has been used for disease-susceptibility locus identification and for a better knowledge of many other processes such as population genetics, due to its relation with immune response and high polymorphic rate [5,6].

Molecular haplotyping is expensive and laborious. On family-based studies, the genotyping of the relatives of each studied subject is required to establish phases. For other population-based studies there are several molecular methods available that allow a correct construction of the haplotypes [5]. Allele-specific polymerase chain reaction (AS-PCR) [7] and somatic cell hybrids [8], are two of the most used molecular methods for an unambiguously determination of haplotypes on relatively small population studies [5]. The use of statistical methods is a less expensive and time-consuming approach for the inference of haplotypes from a large population genotypic dataset [9]. Several statistical methods have been proposed, but it is unclear how accurate each method is for haplotype estimation on HLA genes.

The most commonly used approach, estimating HLA allele and haplotype frequencies, is the expectation-maximization (EM) algorithm [10]. EM algorithms support complex datasets including large number of individuals with ambiguous haplotypes, since they make an initial guess of the haplotype frequencies [5]. EM-based haplotype frequency estimates can accommodate several loci with an arbitrary number of alleles. However, analysis of a large number of loci can result in a exponentially growing computing time. Furthermore, most reports on the use of EM methods have not provided information on the validity of the estimates, nor on the influence on estimation accuracy of population genetic factors, such as departures from Hardy-Weinberg equilibrium (HWE) and the actual haplotype frequency [11]. The Arlequin V3.0 software [12] integrates several basic and advanced methods for population genetics data analysis, using the EM algorithm to estimate maximum likelihood (ML) haplotype frequencies.

Another alternative is the application of Bayesian methods that incorporate prior expectations based upon population genetic principles [13,14]. Using a wide variety of real and simulated data sets it was demonstrated that Bayesian algorithm is robust to the violation of HWE, to the presence of missing data, and to occurrences of recombination hotspots [15]. The program PHASE v2.1.1 [13,14] implements a Bayesian statistical method and partition-ligation for reconstructing haplotypes from population genotype data.

In this study the accuracy of two of the leading methods of computational haplotype inference – EM algorithm based implemented in Arlequin V3.0 and Bayesian algorithm based implemented in PHASE v2.1.1 was compared for a data set composed of 61 unrelated families phase-known HLA haplotypes (HLA-A; HLA-B; HLA-Cw; HLA-DQB1; HLA-DRB1).

## Results

### Identification of the real HLA haplotypes

All the 5 loci and haplotypes were in HWE. The analysis of all pairs of alleles at different loci revealed the presence of 54 haplotypes (Table 1) in complete LD (|D'| = 1). As shown in Table 1, there were 24 pairs of Class I alleles, 8 pairs of Class II alleles and 22 pairs of Class I plus Class II alleles, all with |D'| = 1.

**Table 1:** Pairs of alleles at HLA different loci, with complete LD (|D'| = 1) (n = 61 individuals)

**Class I**	**Class II**	**Class I + Class II**
A*01-B*37	DR*01-DQB*04	A*31-DQB*05
A*02-B*41	DR*03-DQB*02	A*31-DR*01
A*02-B*58	DR*04-DQB*03	A*33-DQB*03
A*03-B*49	DR*07-DQB*02	A*33-DR*11
A*03-B*56	DR*09-DQB*03	B*40-DQB*06
A*11-B*40	DR*10-DQB*05	B*40-DR*13
A*23-B*45	DR*11-DQB*03	B*41-DQB*03
A*26-B*52	DR*12-DQB*03	B*41-DR*13
A*29-Cw*16		B*45-DQB*02
A*33-B*18		B*45-DR*07
A*33-Cw*05		B*49-DQB*03
B*07-Cw*07		B*49-DR*13
B*14-Cw*08		B*51-DR*09
B*15-Cw*03		B*52-DQB*06
B*37-Cw*06		B*52-DR*15
B*40-Cw*02		B*56-DQB*05
B*41-Cw*07		B*56-DR*01
B*45-Cw*06		B*57-DQB*03
B*49-Cw*07		B*57-DR*04
B*51-Cw*14		B*58-DQB*03
B*52-Cw*12		B*58-DR*04
B*56-Cw*01		Cw*12-DQB*06
B*57-Cw*06		
B*58-Cw*07		

One hundred different extended haplotypes were identified (A-B-Cw-DQB-DRB). After splitting the extended haplotypes into Class I (A-B-Cw) and Class II (DQB-DRB), 47 and 18 real haplotypes were observed respectively. Fifty haplotypes with frequencies above 0.01 were found; 15 Class II (Table 2), 21 Class I (Table 3) and 14 extended haplotypes (Table 4).

**Table 2:** HLA Class II haplotype (2 loci) frequencies above 0.01 determined by direct counting and computational methods (n = 61 individuals)

	**Correct Haplotypes**		**Haplotype Frequency**	
		
		**Real**	**Arlequin V3.0**	**PHASE v2.1.1**
1	DQB1*03-DRB1*04	0.164	0.164	0.163
2	DQB1*02-DRB1*07	0.148	0.148	0.146
3	DQB1*05-DRB1*01	0.139	0.139	0.139
4	DQB1*03-DRB1*11	0.123	0.123	0.122
5	DQB1*06-DRB1*15	0.074	0.074	0.074
6	DQB1*06-DRB1*13	0.074	0.073	0.072
7	DQB1*02-DRB1*03	0.074	0.074	0.073
8	DQB1*04-DRB1*08	0.041	0.041	0.041
9	DQB1*05-DRB1*15	0.025	0.025	0.025
10	DQB1*03-DRB1*13	0.025	0.033	0.031
11	DQB1*05-DRB1*10	0.025	0.025	0.024
12	DQB1*05-DRB1*14	0.016	0.016	0.014
13	DQB1*02-DRB1*13	0.016	0.008	0.010
14	DQB1*03-DRB1*12	0.016	0.016	0.016
15	DQB1*03-DRB1*09	0.016	0.016	0.016
		*I*_*F*_	0.992	0.989
		*I*_*H*_	1.000	1.000
		*MSE*	7.3E-06	5.2E-06

**Table 3:** HLA Class I haplotype (3 loci) frequencies above 0.01 determined by direct counting and computational methods (n = 61 individuals)

	**Correct Haplotypes**		**Haplotype Frequency**	
		
		**Real**	**Arlequin V3.0**	**PHASE v2.1.1**
1	A*24-B*27-Cw*02	0.074	0.096	0.053
2	A*02-B*27-Cw*01	0.049	0.074	0.039
3	A*02-B*27-Cw*02	0.033	0.034	0.031
4	A*03-B*27-Cw*02	0.033	0.041	0.040
5	A*03-B*35-Cw*04	0.033	0.041	0.033
6	A*02-B*15-Cw*02	0.025	0.010	0.024
7	A*11-B*35-Cw*04	0.025	0.033	-
8	A*24-B*27-Cw*01	0.025	0.018	0.018
9	A*26-B*27-Cw*02	0.025	0.016	0.024
10	A*29-B*44-Cw*16	0.025	0.025	0.024
11	A*01-B*37-Cw*06	0.016	0.016	0.016
12	A*02-B*07-Cw*07	0.016	0.032	0.025
13	A*02-B*27-Cw*06	0.016	0.016	0.016
14	A*02-B*44-Cw*05	0.016	0.008	0.010
15	A*03-B*07-Cw*07	0.016	-	0.016
16	A*11-B*27-Cw*01	0.016	-	0.007
17	A*23-B*44-Cw*04	0.016	0.016	0.016
18	A*24-B*07-Cw*07	0.016	0.009	-
19	A*26-B*52-Cw*12	0.016	0.016	0.016
20	A*30-B*35-Cw*06	0.016	0.016	0.016
21	A*32-B*27-Cw*01	0.016	-	0.016
		*I*_*F*_	0.906	0.944
		*I*_*H*_	0.923	0.950
		*MSE*	3.7E-05	2.2E-05
	**Incorrect Haplotypes**			
1	A*11-B*14-Cw*08		0.008	0.015
2	A*32-B*27-Cw*07		0.016	-
3	A*02-B*18-Cw*01		-	0.011
4	A*29-B*27-Cw*02		-	0.017

**Table 4:** Extended HLA haplotype (5 loci) frequencies above 0.01 determined by direct counting and computational methods (n = 61 individuals)

	**Correct Haplotypes**		**Haplotype Frequency**	
		
		**Real**	**Arlequin V3.0**	**PHASE V2.1.1**
1	A*24-B*27-Cw*02-DQB1*03-DRB1*04	0.033	0.041	0.036
2	A*02-B*27-Cw*01-DQB1*05-DRB1*01	0.033	0.049	0.040
3	A*24-B*27-Cw*01-DQB1*05-DRB1*15	0.025	0.025	0.020
4	A*24-B*27-Cw*02-DQB1*02-DRB1*03	0.025	0.033	0.013
5	A*03-B*27-Cw*02-DQB1*03-DRB1*04	0.025	0.025	0.018
6	A*26-B*27-Cw*02-DQB1*03-DRB1*11	0.025	0.025	0.021
7	A*26-B*52-Cw*12-DQB1*06-DRB1*15	0.016	0.016	0.015
8	A*29-B*44-Cw*16-DQB1*02-DRB1*07	0.016	0.008	0.006
9	A*30-B*35-Cw*06-DQB1*04-DRB1*08	0.016	0.016	-
10	A*24-B*07-Cw*07-DQB1*06-DRB1*15	0.016	-	-
11	A*23-B*44-Cw*04-DQB1*02-DRB1*07	0.016	0.016	0.016
12	A*11-B*27-Cw*01-DQB1*05-DRB1*01	0.016	-	0.005
13	A*02-B*27-Cw*02-DQB1*03-DRB1*04	0.016	-	0.014
14	A*02-B*15-Cw*02-DQB1*05-DRB1*10	0.016	0.016	0.016
		*I*_*F*_	0.955	0.952
		*I*_*H*_	0.880	0.923
		*MSE*	1.3E-05	1.1E-05
	**Incorrect Haplotypes**			
1	A*01-B*08-Cw*07-DQB1*02-DRB1*03		-	0.015
2	A*02-B*07-Cw*02-DQB1*03-DRB1*04		0.016	-
3	A*02-B*15-Cw*03-DQB1*03-DRB1*01		0.016	-
4	A*02-B*27-Cw*01-DQB1*02-DRB1*07		-	0.011
5	A*02-B*27-Cw*07-DQB1*02-DRB1*03		0.016	-
6	A*03-B*27-Cw*01-DQB1*05-DRB1*01		-	0.015
7	A*03-B*27-Cw*02-DQB1*02-DRB1*07		0.016	0.013
8	A*11-B*14-Cw*08-DQB1*02-DRB1*07		0.016	-
9	A*24-B*35-Cw*04-DQB1*05-DRB1*01		-	0.020
10	A*29-B*27-Cw*02-DQB1*05-DRB1*01		0.016	-
11	A*29-B*27-Cw*16-DQB1*02-DRB1*07		0.016	0.009

### Computational estimation of the HLA haplotypes

The five runs performed with both PHASE v2.1.1 and Arlequin V3.0 gave the same HLA haplotype results, despite using different random sample groups.

### Variable number of loci

**Figure 1:**
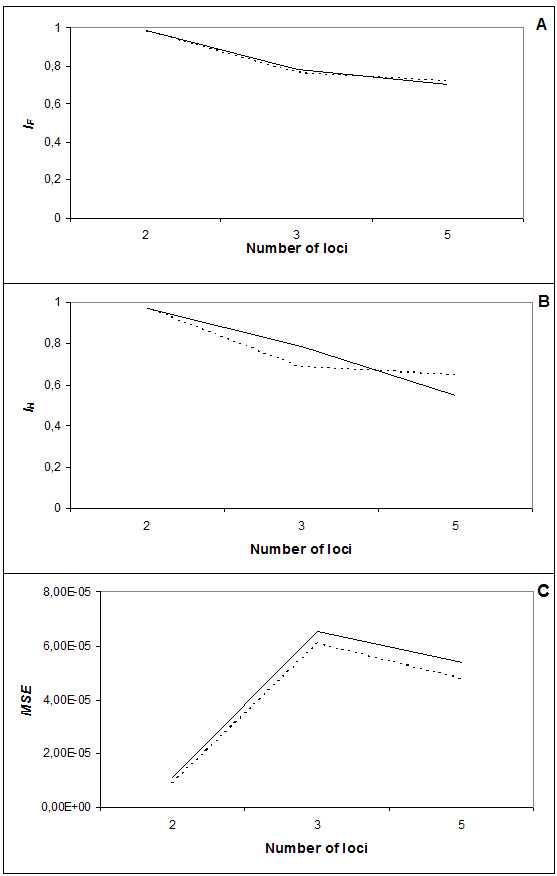
**Influence of the number of loci on haplotype frequency estimation**. A – Similarity index between the frequencies obtained by the used computer packages and the real haplotype frequencies. B – Comparison between the number of different haplotypes identified by the computer packages and the number of different haplotypes obtained by segregation study. C – Overall difference in haplotype frequencies between estimated and true values. The two-locus haplotypes were composed by Class II alleles (DQB1-DRB1), the three-locus haplotypes were composed by Class I alleles (A-B-Cw) and the five-locus haplotypes were the extended haplotypes (A-B-Cw-DQB1-DRB1), all with a sample size of n = 61 individuals. Unbroken line denotes comparisons of Arlequin V3.0 to real data; dotted line, comparisons between PHASE v2.1.1 and real data.

Haplotype frequencies obtained through the family-based study were compared with the results estimated by Arlequin V3.0 and PHASE v2.1.1. Both methods showed an overall decrease in the similarity index (*I*_*F*_) as the number of analyzed loci increased (Fig 1-A). Arlequin V3.0 had the highest *I*_*F *_value for Class II haplotypes (2 loci – 0.988), which was close to its maximal value. PHASE v2.1.1 results showed lower values than Arlequin V3.0, also decreasing as the number of loci increased. However, PHASE v2.1.1 had a higher *I*_*F *_value than Arlequin V3.0, 0.714 and 0.708 respectively (Figure 1-a) when the analysis of five loci haplotypes was performed.

The *I*_*H *_value of both methods for Class II haplotypes was 0.971 (Figure 1-b). Lower results were observed with the three loci haplotypes (PHASE v2.1.1: 0.683; Arlequin V3.0: 0.785). PHASE v2.1.1 registered the highest *I*_*H *_values obtained for the five loci haplotypes.

The mean squared error (*MSE*) increased as the number of loci was increased from two to three. The highest values were obtained with three loci haplotypes, decreasing again with five loci haplotypes (Figure 1-c). The same figure shows that *MSE *values for Arlequin V3.0 were always higher than PHASE v2.1.1, and that the discrepancy between these methods was more evident as the number of analyzed loci increased.

### Variable number of samples

**Figure 2:**
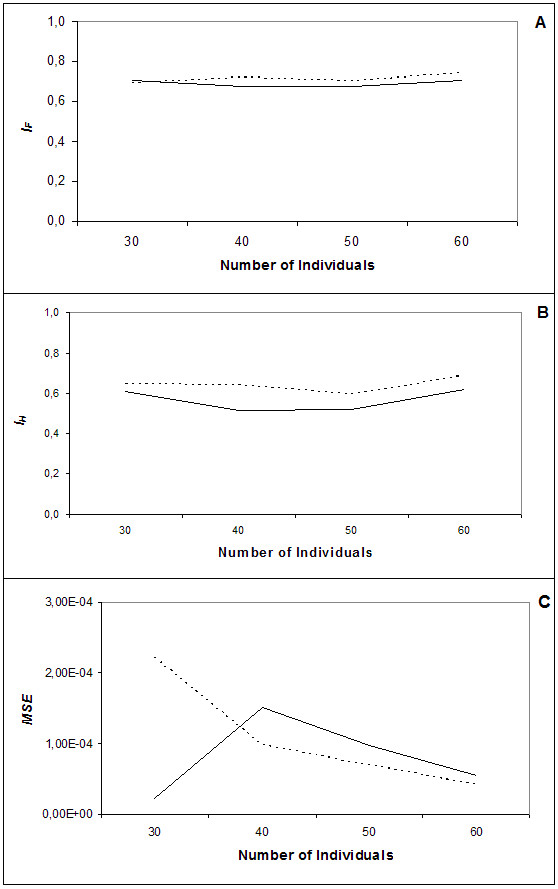
**Influence of sample size on haplotype frequency estimation**. A – Similarity index between the frequencies estimated by the computer packages and the real haplotype frequencies. B – Comparison between the number of different haplotypes estimated by the computer packages and the number of haplotypes obtained by segregation. C – Overall difference in haplotype frequencies between estimated and true values. Unbroken line denotes comparisons of Arlequin V3.0 to real data; dotted line, comparisons between PHASE v2.1.1 and real data.

In order to investigate the performance of both methods with different sample sizes, four different randomly selected groups of samples (30, 40, 50 and 60 samples) with different sizes were defined for each one of the five runs performed. These groups of samples were investigated with both methods, and the results of the estimation accuracy measures are shown in Figure 2.

Despite increasing the number of samples, both methods exhibited a small range of variation in the *I*_*F *_value (Figure 2-a). Arlequin V3.0 showed the same *I*_*F *_with 30 or 60 samples (0.709), although a small reduction of this value was obtained with 40 or 50 individuals (0.675). Different results were obtained with *I*_*H *_Arlequin V3.0 values (Fig. 2-b). *I*_*H *_decreased when the sample size was increased from 30 to 40 individuals (0.611 to 0.517), increasing again when the test was performed with 40 to 60 individuals (the highest difference was obtained from 50 to 60 – 0.523 and 0.620 respectively).

PHASE v2.1.1 analysis reported smaller *I*_*F *_values than Arlequin V3.0 with 30 samples (0.690), but after increasing the sample size the values obtained were always higher than Arlequin V3.0 (Figure 2-b).

The *MSE *of Arlequin V3.0 for 30 samples was 1 order of magnitude lower than the registered by PHASE v2.1.1 (Figure 2-c). When the sample value was increased from 30 to 40 individuals the Arlequin V3.0's *MSE *increased from 2.1E-05 to 1.5E-04, while in PHASE v2.1.1 this value decreased from 2.2E-04 to 9.8E-05. Both methods showed an overall decrease when samples where higher than 40 individuals, although PHASE v2.1.1 had the best *MSE *values.

### Haplotypes with frequencies above 0.01

There was 100% concordance between real Class II haplotype phases (2 loci) and those predicted computationally by both methods (Table 2). The *I*_*F *_values were 0.992 for Arlequin V3.0 and 0.989 for PHASE v2.1.1, which were both close to its maximal value. Within the two loci haplotype analysis, PHASE v2.1.1 *MSE *value was lower than Arlequin V3.0, despite the maximum *I*_*H *_value reported by both methods.

*I*_*F *_results were lower with Arlequin V3.0 than PHASE v2.1.1 (0.906 and 0.944 respectively) for the 3 loci haplotypes (Class I). Although Arlequin V3.0 generated 18 out of 21 real Class I haplotypes, it also created 2 incorrect haplotypes with frequencies above 0.01 (Table 3). PHASE v2.1.1 was more effective and accurate in haplotype construction, as well as in frequency estimation, thus presenting the best *MSE *value of the two methods, as shown on Table 3. Despite having a better *MSE *value (Table 3), PHASE v2.1.1 generated 3 incorrect haplotypes, one more than Arlequin V3.0.

Regarding the 5 loci haplotypes (Table 4), PHASE v2.1.1 had a more favourable *MSE *(1.1E-05, versus Arlequin V3.0's 1.3E-05), and a better performance in *I*_*H *_(0.923, versus Arlequin V3.0's 0.880). Moreover, PHASE v2.1.1 presented 6 incorrect 5 loci haplotypes (with frequencies above 0.01), one less than Arlequin V3.0. The *I*_*F *_value was similar between Arlequin V3.0 and PHASE v2.1.1 (0.955 and 0.952 respectively) showing that both methods had a high accuracy on extended haplotypes estimation (Table 4). One of the incorrect haplotypes generated by PHASE v2.1.1 showed a frequency of 0.02, but in general this software estimated lower frequencies for the incorrect haplotypes that were generated by both methods. In this group of haplotypes (5 loci) the number of possible combinations between the alleles of the various loci increased due to the increase of the polymorphic rate but the number of real haplotypes decreased when compared to the 3 loci haplotypes with frequencies above 0.01.

## Discussion

We have tested the effectiveness and accuracy of two computational algorithms to estimate haplotype frequencies and to predict haplotype phases using real HLA haplotypic data. To our knowledge this was the first work aiming to test statistical methods using haplotypic data from the HLA region, characterized by a high rate of polymorphic sites and LD.

Contrasting with the findings of Xu et al. [16], the decrease in accuracy of both methods occurred when LD rate between the alleles increased. Both methods registered a higher decrease in the *I*_*F *_value from 2 to 3 loci than from 3 to 5 loci (Figure 1-a), suggesting that the presence of HLA-B was the main factor influencing the algorithms' performance. This finding was confirmed when the number of individuals varied within the 5 loci haplotypes (A-B-Cw-DQB-DRB) (Figure 2).

Regarding the variable number of loci analysis, it is relevant to notice that the 3 loci and 5 loci haplotypes included the locus HLA-B which has the highest level of polymorphism within the HLA region [1]. Moreover being especially polymorphic, HLA-B alleles were present in 39 of the pairs of alleles with |D'| = 1.

The *I*_*F *_and *MSE *results show similar behaviour in both methods in terms of overall haplotype frequency estimation, despite the variation on the number of tested loci. These findings are in contrast to those observed by other authors [14,17], but are quite similar to the results obtained by Zhang et al. [18]. Despite the differences between the EM-based method and PHASE v2.1.1 observed by the first authors [14,17], the work of Zhang et al. [18] revealed that, in general, for most populations, there is no significant differences between the PHASE v2.1.1 method and the EM-based method when comparing estimated and true sample haplotype frequencies.

The low variability observed in Arlequin V3.0 *I*_*F *_values when different numbers of samples were tested is in agreement with previous reports performed with EM algorithms in haplotype frequency estimation in other genes [11,16]. These previous studies demonstrated that the EM-based method has a very good performance under a wide range of population and data set scenarios.

Analyzing the *MSE *values of variable number of samples and loci, PHASE v2.1.1 had the best performance, exhibiting higher *MSE *values than Arlequin V3.0 only in the smallest group of samples (Figure 2-a).

Concerning haplotypes with frequencies above 0.01, Arlequin V3.0 *I*_*H *_values had a progressive decline as the number of loci increased. The decrease in haplotype frequency accuracy in Arlequin V3.0 might be the result of the presence of a high polymorphic site, like HLA-B, as well as the increase in the number of loci (from 2 to 3 and to 5 loci) with variable number of polymorphic sites. The *I*_*H *_decrease, registered by PHASE v2.1.1 (haplotypes with frequency above 0.01), was very small when the number of loci was increased from 3 to 5, suggesting once more that in PHASE v2.1.1 the presence of HLA-B, and consequently the increase of the polymorphic rate in the larger extended haplotypes, was the major factor influencing its performance.

Like the *I*_*F*_, the *I*_*H *_values in both methods seem to be influenced by the number of polymorphic sites and the rate of pairs of alleles in complete LD.

The best results in both algorithms were obtained when only the Class II real haplotypes with a frequency above 0.01 were analysed. The Class II region has a low polymorphic rate when compared with the Class I region, decreasing the number of possible combinations within the alleles. However, this group has less pairs on complete LD (Table 1), which could suggest a low effectiveness in both methods [16,19,20]. No incorrect haplotypes were estimated, suggesting that for this type of samples both Arlequin V3.0 and PHASE v2.1.1 had a very good performance. The results obtained with Class II were in concordance with the work of Adkins [21] in single nucleotide polymorphism (SNP) haplotypes. This author showed that both EM-based methods and PHASE could identify all the haplotypes with a frequency above 0.01.

The increase of polymorphic rate in the 3 loci haplotypes (Class I) increased the number of different real haplotypes with frequencies greater than 0.01. One incorrect haplotype was generated in common by both methods, presenting a lower frequency when calculated by Arlequin V3.0. The presence of this haplotype (A*11-B*14-Cw*08) in both methods might be caused by the complete LD between B*14 and Cw*08 (Table 1). Higher haplotypic frequencies (above 0.01) increase the confidence and the accuracy of computational methods on haplotype determination. Consequently, it decreases the probability of identifying an incorrect haplotype.

The strong LD among the markers in this region is well known [6], and was confirmed by the results displayed on Table 1. Despite the influence of LD in the performance of computational methods [16,19,20], the results demonstrated that the rate of polymorphic sites within the loci also plays an important role. The increase of LD rate was responsible for the decrease of the accuracy of these programs contrary to the findings of other authors, whose research addressed other genetic regions with both high and low LD [16,19,20]. In these studies, the authors worked with SNP where the probability of recombination events is small despite the existing LD rate. The results obtained in the present work might be a consequence of HLA system specificities. The HLA high rate of heterozygosity allows a large number of combinations between different alleles [2], even with the strong LD in this region. Therefore it is likely that in both methods the decrease of accuracy when the LD rate increases is the result of the high variability in pairs of alleles with |D'| = 1.

The algorithm of PHASE v2.1.1 is similar to the algorithms of Stephens et al. [14] and Stephens and Donnelly [13], which contains a "pseudo-Gibbs sampling step" [22]. It should be noted that the theoretical convergence of the underlying algorithm of PHASE v2.1.1 remains an open problem [22], although the comparisons of Stephens and Scheet [22] showed that, on average, PHASE v2.1.1 produces more accurate haplotype estimates (by two different measures) than does the algorithm of Stephens and Donnelly [13]. To remedy this problem, Zhang et al. [23] developed a novel coalescence-guided hierarchical Bayesian method wich uses a hierarchical structure to directly model the coalescence relationship among modern-day haplotypes. This method has shown to have merits compared with PHASE v2.1.1.

## Conclusion

This work suggests that high HLA haplotype frequency in the population under study is an important factor for the accuracy and performance of Arlequin V3.0 and PHASE v2.1.1, despite the presence of a high rate of polymorphic sites and alleles with complete LD.

Arlequin V3.0 had the best performance on haplotype inference and frequency estimation when dealing with less than 5 loci haplotypes. PHASE v2.1.1 had the best overall performance both in haplotype prediction and in frequency calculation. Nevertheless, in general and like reported in previous works [18], the difference found in this work between PHASE v2.1.1 and Arlequin V3.0 was insubstantial.

The best results of both algorithms were obtained when only real haplotypes with a frequency above 0.01 were analysed.

From a practical point of view, both methods provide advantages and disadvantages to estimate HLA haplotypes. Arlequin V3.0 main advantage may be the format of the input file, which does not require any transformation on the haplotypic data and supports the HLA nomenclature currently used. With PHASE v2.1.1, the haplotypic data needs to be transformed into a numeric code, prior to running the program, and the results have to be transformed again to the original HLA nomenclature. Hence, data transformation can become very laborious when using large haplotypic data sets. PHASE v2.1.1 main advantage is the fact that all the possible haplotypes are displayed in the output file, which can be useful in certain studies. This does not happen with Arlequin V3.0 where only the most frequent haplotypes (above a frequency established by the program) are displayed on the output file.

The present work indicates that, like with other genes and sample sizes [5,11,16,18-21], computational methods can provide an effective calculation of HLA haplotype frequencies by using data from unrelated individuals. These computational methods can provide an accurate prediction of haplotype phases on this particular region, despite the strong LD and high polymorphism within alleles from different HLA loci. This work may present some useful information about statistical approach in studies using HLA.

## Methods

### DNA Samples/Genotyping

Blood was collected from 61 probands (38 males and 23 females) and their relatives (228 individuals; 107 males and 121 females) belonging to unrelated families, under informed consent. DNA extraction was performed by the salting-out technique [24]. HLA typing was carried out for the Class I loci: HLA-A, HLA-B, HLA-Cw; and Class II loci: HLA-DQB1, HLA-DRB1, by Polymerase Chain Reaction – Sequence Specific Primers (PCR-SSP), as described by Olerup and Zetterquist (1992) [25] using the *Olerup *SSP HLA typing kit (*Olerup *SSP AB, Sweden).

### Real haplotypes identification

The HLA A-B-Cw-DQB1-DRB1 real haplotypes were determined through a family-based study using family members of the 61 probands. A segregation study of the HLA haplotypes was performed on the probands pedigree, allowing the identification of each individual real haplotypes.

### Hardy-Weinberg equilibrium (HWE)

The HWE was calculated locus by locus and for whole haplotype, using the software package GENEPOP v3.4 [26]. The Markov chain approximation was used with 100000 steps and 1000 dememorization steps definition.

### Pairwise LD between alleles

LD, between pairs of alleles at different loci, was calculated through the computing of the standardized LD value (D') [27]. D' is the normalization of the LD, dividing it by the theoretical maximum value for the observed allele frequencies ($D'=\frac{LD}{LD\max}$). |D'| = 1 indicates complete LD and D' = 0 corresponds to total absence of LD.

### Haplotype computational estimation

Two different algorithms were used to examine the accuracy of computational haplotype inference. PHASE v2.1.1 was run with the default options, with an exception: the number of iterations of the final run of the algorithm was increased to five restarting points (-X option). Arlequin V3.0 was run using the following settings: EM algorithm performed at the haplotype level, ε = 1e-7. 5 significant digits for output, 50 starting points for EM algorithm and a maximum of 1000 iterations. Both programs were runned five times, for each of the used scenarios (variable number of individuals and variable number of loci). New input data files were created for every run. Each data file was composed by different randomly selected sample groups.

### Measures of estimation accuracy

The mean squared error (MSE) [11] was used to measure the accuracy of computational algorithms in haplotype frequency estimation. The MSE incorporates all the *k *haplotype frequencies reflecting the overall difference in haplotype frequencies between estimated and true values for a particular data set:

$$MSE=\sum_{k=1}^{h}(p_{ek}-p_{tk}{)}^{2}/h$$

where *h *is the number of haplotypes in the data set, *p*_*ek *_and *p*_*tk *_are the estimated and real (in this case) haplotype frequencies for the *k *haplotype. Another two measures, *I*_*F *_(Similarity Index) and *I*_*H *_(Haplotype Identification Index) [10], were also used to estimate the efficiency of computational algorithms. *I*_*F *_ranges from 0 to 1 (the value 1 means that the real and the estimated frequencies are identical), and measures the haplotype frequency estimations, describing how closely the estimated haplotype frequencies are to the real frequencies. This index is defined as the proportion of haplotype frequencies which are in common between estimated and true frequencies:

$$I_{F}=1-\frac{1}{2}\sum_{k=1}^{h}\left|p_{ek}-p_{tk}\right|$$

where *h*, *p*_*ek *_and *p*_*tk *_are defined as above. *I*_*H *_measures the accuracy of haplotype identification, comparing the number of different haplotypes obtained experimentally with the number of different haplotypes identified by the computer software. A haplotype is defined as being detected if it has an estimated frequency of at least 1/(2n) in a population of n individuals [10]. *I*_*H *_is defined as:

$$I_{H}=\frac{2\left(m_{true}-m_{missed}\right)}{m_{true}-m_{est}}$$

where *m*_*true *_is the number of haplotypes known to occur, *m*_*est *_is the number of inferred haplotypes with frequency ≥ 1/(2n), and *m*_*missed *_is the number of known haplotypes that were not inferred.

## Authors' contributions

BFB participated in the design of the study, performed the statistical analysis and drafted the manuscript. MRS carried out the determination of real haplotypes. RNF participated in the statistical analysis. ARC participated in the design of the study and helped to draft the manuscript. MJP participated on the determination of real haplotypes. JPP carried out genotyping assays. HS helped to draft the manuscript. MGM participated in the design of the study. CS participated in the design of the study and helped to draft the manuscript. AB participated in the design of the study. JBA conceived the study and participated in its coordination and helped to draft the manuscript. All authors read and approved the final manuscript.
